# Antibacterial and Antifungal Therapy for Patients with Acute Pancreatitis at High Risk of Pancreatogenic Sepsis (Review)

**DOI:** 10.17691/stm2020.12.1.15

**Published:** 2020

**Authors:** V.G. Firsova, V.V. Parshikov, M.V. Kukosh, A.S. Mukhin

**Affiliations:** Surgeon, City Hospital No.35, 47 Respublikanskaya St., Nizhny Novgorod, 603089, Russia; Professor, Department of Hospital Surgery named after B.A. Korolyov, Privolzhsky Research Medical University, 10/1 Minin and Pozharsky Square, Nizhny Novgorod, 603005, Russia; Professor, Department of Faculty Surgery and Transplantology, Privolzhsky Research Medical University, 10/1 Minin and Pozharsky Square, Nizhny Novgorod, 603005, Russia; Professor, Head of the Department of Hospital Surgery named after B.A. Korolyov, Privolzhsky Research Medical University, 10/1 Minin and Pozharsky Square, Nizhny Novgorod, 603005, Russia

**Keywords:** acute pancreatitis, sepsis, antibiotic therapy, antibiotic prophylaxis, antifungal therapy

## Abstract

Controlling infection is crucial in treating patients with acute pancreatitis (AP). The infectious process in AP often predisposes to subsequent sepsis by damaging not only the pancreas, but retroperitoneal tissues as well. Among other AP-associated factors, are the rapidly developing immune imbalance, the poor penetration of antimicrobial agents into necrotic tissue, and the impossibility of a single surgical debridement. Antibacterial and antifungal therapy for patients with infected necrosis and AP-associated extra-pancreatic infections remains a complex and largely unresolved problem, partially due to the high occurrence of multiresistant pathogens. The preventive use of antimicrobial agents has been discussed in the literature; however, the lack of consistent results makes it difficult to develop a unified strategy and clinical guidelines on this specific issue. Recent meta-analyses provide no conclusive evidence that antibacterial prophylaxis reduces the infection rate, mortality, or the need for surgical treatment in patients with necrotizing pancreatitis. We found only two studies indicating the benefits of using carbapenems for prophylactic purposes and one meta-analysis indicating a reduction in mortality under antibiotic treatment started no later than 72 h after the onset of the attack. Selective bowel decontamination is considered as one of the preventive anti-infection measures, although the available data may not be fully reliable.

The main indications for antibacterial therapy in patients with AP are confirmed infected necrosis or extra-pancreatic infection, as well as clinical symptoms of suspected infection. Intra-arterial administration or local treatment with antibiotics can increase the efficacy of antibacterial therapy. No randomized studies on antifungal prophylaxis in AP are available; some reports though recommend using such therapy among patients at high risk of invasive candidiasis.

## Introduction

Infection is a life-threatening factor in patients with acute pancreatitis (AP), as evidenced by significantly higher mortality rates in this category of patients as compared with those with sterile necrosis [1–6]. The infectious process in AP often predisposes to subsequent sepsis by damaging not only the pancreas, but retroperitoneal tissues as well. Among other AP-associated pathogenic factors, are the rapidly developing immune imbalance, the poor penetration of antimicrobial agents into necrotic tissue, and the impossibility of a single surgical debridement [7, 8]. These factors provide a rationale for the use of antibacterial agents to prevent bacterial contamination of necrotic foci; however, the efficacy, timing, and choice of optimal medications remain the subject of discussion [9, 10]. As reported, using antibacterial agents in patients with AP is a common clinical practice all over the world. There is no doubt though that the early and long-term use of antibiotics can provoke intestinal dysbiosis with subsequent dissemination of multiresistant strains of bacteria and fungi, including those that make up normal human microbiota [11, 12]. The current concepts and evidence concerning this problem have earned special attention in the literature [13–15].

## Forms and complications of acute pancreatitis

In the revised international guidelines from the Acute Pancreatitis Classification Working Group (2012), two forms of AP are discerned: interstitial and necrotic. Necrotic pancreatitis can manifest as pancreatic, peripancreatic or both; each of these forms can be either sterile or infected.

Complications of AP are divided into local and systemic. Local complications include pancreatogenic fluid collections, compression of the gastric outlet, thrombosis of the portal or splenic veins, necrosis of the colon wall, etc. The systemic complications manifest by worsening of concomitant diseases.

In the clinical picture of AP, two overlapping phases can be distinguished: early (a first week from the onset of the attack) and late (weeks and months from the onset).

Local complications of interstitial pancreatitis include acute peripancreatic fluid collection and pancreatic pseudocyst. In the case of necrotizing pancreatitis, there is acute necrotizing collection and walled-off necrosis.

The course of AP involves transient (lasts no longer than the first 48 h) and persistent (sustains over 48 h) organ failure that affects the respiratory, cardiovascular or renal systems. The diagnosis of mild AP is made in the absence of organ failure, local or systemic complications; moderate AP includes cases with transient organ failure and/or local or systemic complications; severe AP encompasses all cases with persistent organ failure [16].

In contrast to the international classification, the Russian Society of Surgeons (RSS) does not discern local complications between the necrotic and interstitial forms of the disease. According to the RSS classification, local complications include peripancreatic infiltrate (with no clear distinction from surrounding tissues, up to 4 weeks from the onset of the disease) and pancreatic pseudocyst (with an apparent wall, after 4 weeks from the onset); if these formations become infected, purulent-necrotic parapancreatitis and pancreatic abscess (respectively) ensue. The first two weeks from the onset of AP are considered the early phase of the disease; the late phase extends over the following weeks or months. The mild form of AP does not involve pancreatic necrosis or organ failure. Clinical cases with local complications or transient organ failure are diagnosed as moderate AP. In severe AP, the development of purulent necrotic parapancreatitis or persistent organ failure are of major diagnostic relevance [17].

According to the Third International Consensus Definitions for Sepsis and Septic Shock (2016), sepsis is defined as life-threatening organ dysfunction caused by an unregulated (inadequate) response to infection. The diagnosis of infection-induced organ dysfunction is made according to a sudden change of ≥2 in the total SOFA score [18].

According to the criteria of the European Centre for Disease Prevention and Control, multidrug-resistant microorganisms are considered as such if they are resistant to at least one drug out of three classes of antibacterial agents. The “extremely resistant” term pertains to microorganisms sensitive to no more than two classes of antibiotics [19].

## Preventive use of antibacterial agents in acute pancreatitis

A number of studies [20–27] analyze the results of preventive use of antibacterial agents in patients with AP. They weigh the frequency of infection, development of sepsis, need for surgical treatment, and mortality rate (Table 1). According to these indicators, quite a few studies demonstrate no significant improvement under antibiotic prophylaxis [12, 21–24, 28, 29].

**Table 1:** Meta-analyzes of reports on the prophylactic use of antibacterial drugs in acute pancreatitis

Authors	Nature of the study	Results
Dambrauskas, et al., 2007 [20]	10 RCTs, 1279 patients with necrotic AP, additional analysis by drug classes (cephalosporins, fluoroquinolones, carbapenems)	Carbapenems, but not other antibiotic groups, reduce the incidence of necrosis infection and sepsis, and also diminish the need for surgical treatment
Bai, et al., 2010 [21]	9 RCTs, 519 patients with necrotic AP	No reduction in mortality or necrosis infection rate
Villatoro, et al., 2010 [22]	7 RCTs, 404 patients with necrotic AP, additional analysis by drug classes (beta-lactams, fluoroquinolones, imipenem)	No reduction in mortality, incidence of necrosis infection or extrapancreatic and fungal infections; no change in the need for surgical interventions Imipenem significantly reduces the incidence of pancreatic infection
Wittau, et al., 2011 [23]	14 RCTs, 841 patients with severe AP	No decrease in mortality, the incidence of necrosis infection, extrapancreatic infections, or frequency of surgical interventions
Jiang, et al., 2012 [24]	11 RCTs, 622 patients with severe AP	No overall reduction in mortality, but cases of increased survival of specific categories of patients were observed
Rada, et al., 2014 [25]	19 RCTs	May reduce mortality and duration of hospital stay; low level of evidence
Lim, et al., 2015 [26]	11 RCTs, 864 patients with necrotic AP: group 1: RCTs + cohort studies group 2: RCTs only group 3: cohort studies only	No reduced frequency of necrosis infection, fungal infections, or surgical interventions. Mortality decreased in groups 1 and 3
Ukai, et al., 2015 [27]	6 RCTs, 397 patients with necrotic AP Treatment started no later than 72 h after the onset of the attack or no later than 48 h after hospitalization	Significantly lower mortality rates and reduced incidence of infected necrosis

Note: RCTs — randomized cohort studies.

In two meta-analyses [25, 26], a conclusion was made on decreased mortality in patients with prophylactic use of antibacterial drugs; one of these reports, in addition to randomized cohort studies (RCTs), includes cohort studies as well. However, these studies have limitations that are essential for understanding and applying their results. The RCTs, which formed the basis of meta-analyzes, included a relatively small number of patients (no more than 114); only part of the quoted studies was double-blind, placebo-controlled and with a specific reference to patients with severe AP. A significant number of patients in the control groups received antibiotics at a later period, and no separate accounts for mortality were made. Notably, the course of infection depends not only on antibiotic therapy, but also on a combination of interrelated factors (the adequacy of infusion and nutritional support, the immunological status of the patient, the need for surgical interventions, the duration of intestinal paresis, the start of enteral nutrition, etc.).

Comparison of different classes of antibiotics (cephalosporins, fluoroquinolones, carbapenems) in the context of their prophylactic efficacy in AP revealed a significant decrease in the incidence of infected necrosis (but not incidence of sepsis), the need for surgical treatment, and mortality only with carbapenems: those were imipenem and meropenem in meta-analysis [20], and imipenem in study [22].

The meta-analysis of Ukai et al. [27] indicates the importance of timing for the preventive use of antibiotics: a significant decrease in the incidence of infection and mortality is achieved when antibacterial drugs are used no later than 72 h after the onset of the attack.

Overall, the studies on the prophylactic use of antibacterial drugs in AP did not produce consistent results and can be characterized by a medium or low level of evidence. This situation is reflected in practical recommendations set out in the guidelines from various countries [17, 30–33] (Table 2). Therefore, scientists continue searching for criteria to identify patients with the highest risk of infection, in a hope to clearly demonstrate the feasibility of antibacterial prophylaxis. In [34], the authors proposed two independent risk factors to identify infected necrosis in patients with severe AP: those were an increase in intra-abdominal pressure above 13 mm Hg and a serum level of D-dimer of ≥933 μg/L during the first three days of the disease; these predictors had a sensitivity and specificity of 90 and 58%, and 95 and 58%, respectively. Chen et al. [5] showed that in order to predict infected necrosis, and not only to assess the severity of AP, one can use the values of maximum hematocrit (≥50%), urea (≥8.42 mmol/L), C-reactive protein (≥257 mg/L), and procalcitonin (≥1.39 ng/ml) obtained during the first 48 h of the disease. The hematocrit and C-reactive protein values are characterized by relatively low sensitivity and high specificity (56 and 73%, 45 and 89%, respectively); therefore, in the absence of high values of these indicators, the likelihood of developing infected necrosis is low. The significance of serum levels of D-dimer (as an independent risk factor for infected necrosis) was not confirmed in this large-scale study. The authors’ data [5] are consistent with the results of other works [35–38]. With the combined use of all four of the above parameters in predicting the development of infected necrosis, a sensitivity of 68% and a specificity of 77% could be reached [5].

**Table 2:** Recommendations on the prophylactic use of antibacterial drugs for acute pancreatitis in various countries

Sources	Contents of recommendations for the prophylactic use of antibiotics	Strength of recommendation, level of evidence
Russian national clinical guidelines, 2015 [17]	Not recommended in AP of moderate severity In severe AP — not recommended for the first 3 days 2^nd^ week of the disease — systemic antibiotic prophylaxis (III–IV generation cephalosporins or II–III generation fluoro-quinolones in combination with metronidazole; carbapenems as reserve drugs)	Level of evidence — C
International Association of Pancreatology and American Pancreatic Association, 2013 [30]	Intravenous antibiotic administration is not recommended	Strength of recommendation — 1 Level of evidence — B
American College of Gastroenterology, 2013 [31]	Not recommended	Strength of recommendation — 1 Level of evidence — B
Japanese Guideline, 2015 [32]	Prophylactic antibiotics for severe AP and necrotizing pancreatitis can improve prognosis if performed within 72 h from the onset of the disease	Strength of recommendation — 2 Level of evidence — B
American Gastroenterological Association Institute Clinical Guidelines Committee [33]	Prophylactic antibiotics for AP are not recommended	The recommendation requires a strictly individual approach to the patient Low level of evidence

A decrease in the absolute number of lymphocytes in the peripheral blood within 48 h from the onset of AP reflects immunosuppression that occurs in the early phase of the disease. The threshold level of lymphocytes ≤0.66**·**109/L indicates a high probability of developing infected necrosis with a sensitivity of 83.7% and a specificity of 66.7% [39].

In a study by Zeng et al. [40], the following predictors of infected necrosis were used: an increased level of lactate dehydrogenase, severe AP as per CT, a late start of infusion therapy, and hypoxemia.

According to Garret et al. [41], multiple organ failure in the early phase of AP and portosplenomesenteric venous thrombosis can serve independent risk factors for infected necrosis. Other researchers point to the association of such thrombosis with the probability of infection, although the linking mechanisms of pathogenesis remain unclear [42]. The occurrence of organ dysfunction requires more “aggressive” tactics, including catheterization of the central vein or bladder, and in some cases invasive ventilation, all of which increase the risk of additional infections [29].

Most often, early multiple organ failure occurs in patients with extended necrosis, which is another risk factor for infection [43, 44]. The high incidence of pancreatic infection in patients with extended necrosis, especially in combination with dynamic intestinal obstruction in the early phase of the disease, is also indicated by Moran et al. [45]. According to study [46], a high APACHE II score and hypotension during the first week of AP are good predictors of infected necrosis.

Another factor associated with a high probability of pancreatic infection is the biliary etiology of AP. The pathogenesis of biliary AP involves obstruction of duodenal papilla resulting in biliary and pancreatic hypertension. Acute obstruction causes damage to hepatocytes, disruption of the enterohepatic bile circulation and the rapid development of cholangitis, which contributes to the infection of necrosis zones in the pancreas and parapancreatic tissue [47].

Most scientists believe that the leading mechanism of infection in AP is the translocation of bacteria through the intestinal wall into the systemic circulation, which corroborates with the predominance of intestinal flora in primary cultures from necrotic foci and the results of 16S RNA sequencing obtained from the patients’ blood samples [5, 48–51]. Bacterial translocation is defined as a process in which bacteria or bacterial antigens (lipopolysaccharides, peptidoglycans), normally present in the GI lumen, cross the intestinal barrier and penetrate into otherwise normal tissue, where they can cause an infection process or activate the immune response thus leading to subsequent organ dysfunction and failure [52].

At present, the intestine is viewed as playing an important role in the processes of systemic inflammation, sepsis, multiple organ failure, and progression of AP [53–56]. Damage to the intestinal barrier in AP is caused by disturbances in microcirculation resulted from the increasing intestinal pressure, shock, and the occurrence of microthrombosis [57]. These events prompted the use of antibacterial drugs for selective intestinal decontamination (SID), aiming at suppressing potentially pathogenic microorganisms (e.g., gram-negative bacteria, methicillin-sensitive *Staphylococcus aureus*, yeasts) in favor of commensal anaerobic bacteria [52, 58, 59]. In the classic version, SID includes [60]:

1. a short course (4 days) of parenteral antibiotics to control endogenous infections caused by potentially pathogenic microorganisms in the patient’s body at the time of admission;
2. in the ICU, non-absorbable antibiotics (for example, a combination of polymyxin E, tobramycin, amphotericin B) *per os* to control secondary endogenous infections caused by bacteria from the oropharynx or intestines during treatment;
3. a high level of hygiene that prevents exogenous infection;
4. bacterial monitoring (oral cavity, rectum) twice a week to monitor the results of SID and identify resistant microorganisms in the early stages.

Just a few studies reported the efficacy of SID in patients with AP; of those, only one [61] was a randomized controlled trial. The authors concluded that SID caused a significant decrease in mortality and complications in patients with severe AP. However, these results should be taken with the understanding that other systemic antibiotics (apart from the SID scheme) were given to non-randomized patients in the study. As a note of caution, SID can contribute to the selection of multiresistant strains in some patients, especially those staying in the ICU for a long time; therefore, careful microbiological control during the implementation of SID is advised [60, 62–64]. Currently, many authors admit the usefulness of SID in patients with severe AP, but further research in this area is required to increase the level of evidence [30, 43, 58, 65]. In recent international practical manuals, recommendations on SID in patients with AP are not found [17, 32, 33].

## Antifungal prophylaxis for acute pancreatitis

In primary bacterial cultures from foci of infected necrosis, fungi are identified in 6–46% of patients with AP [3, 51, 66–70]. This unusually wide range of the results is due to the different assessments (either per the entire population of patients with AP, or among patients with necrotic pancreatitis, or those with severe AP). Another factor contributing to this inconsistent data is the difficulty of discerning between candida colonization and invasive candidiasis, which is due to some diagnostic imperfection [71]. In patients receiving antibiotic therapy in the stage of walled-off necrosis (after 4 weeks from the onset of the disease), the frequency of fungal infection reaches 27% of patients against the background of antibiotic therapy [72]. According to Hall et al. [73], the proportion of patients treated in the ICU for severe AP complicated by invasive candidiasis is 18%. Among various species of candida, *Candida albicans* prevails [67, 73, 74].

In patients with severe AP, there is a combination of several risk factors for fungal infection: the presence of a central venous catheter, complete parenteral nutrition, and the use of broad-spectrum antibiotics [75] (although a meta-analysis [26] did not find a link between systemic antibiotic therapy and fungal infections). The rationale for the antifungal preventive measures stems from the increased rates of complications and mortality associated with candida invasive infection, the extreme difficulties of removing the fungi from a poorly perfused, partially necrotic pancreas, and the chance to delay surgery by including antifungal drugs in the treatment regimen [70, 74, 76]. However, to date, no large-scale RCTs on preventing of fungal infection in necrotic AP have been conducted; therefore, the significance of this therapy is not clearly determined.

In the recently revised practical guide from Japan [32], the routine use of antifungal prophylaxis in patients with AP is not recommended. In the Russian Society of Surgeons guidelines, the manuals of the International Association of Pancreatology, the American Pancreatic Association, and the American Gastroenterological Association, there are no specific instructions on that subject [17, 30, 33]. In a number of studies, attempts were made to identify patients (among surgical patients with severe AP) at high risk of developing invasive candidiasis. This group of high-risk patients may benefit from antifungal prophylaxis and have a better survival rate, while its implementation in patients with a low risk of invasive candidiasis can lead to the development of multiresistant fungal strains [73]. To solve the problem, it was proposed to use special score systems (indices), in particular: the Candida Score (CS), the Modified Invasive Candidiasis Score (MICS), and the Candida Colonization Index Score (CCIS). The CS indices are calculated as follows: total parenteral nutrition, surgical intervention, multifocal colonization — 1 point, severe sepsis — 2 points. The MICS index takes into account any use of systemic antibiotics or the presence of a central venous catheter in combination with at least two of the following factors: complete parenteral nutrition, dialysis, “large” surgical intervention, and the use of glucocorticoids or immuno-suppressants. The CCIS index is defined as the ratio of the number of zones of colonization by candida (oral cavity, tracheobronchial tree, urinary tract, a discharge from the abscess drainage) to the total number of examined zones. In the work of Hall et al. [73], these indices were measured in patients with severe AP and found to have a low sensitivity (<70%) and a low positive predictive value (<50%), but a high negative predictive value (72, 85 and 91% for CS, MICS, and CCIS, respectively); the CS index was the most specific (85%). According to these authors, with a CCIS value <0.5, there is a low probability of having invasive candidiasis; therefore, antifungal therapy is indicated only with CCIS >0.5. Other researchers believe that the CCIS >0.5 cannot serve a threshold triggering the antifungal prophylaxis because such CCIS scores are observed in more than 25% of patients in the ICUs; this indication may lead to excessive use of antifungal drugs and change the ecology and drug sensitivity of the candida [77, 78]. However, the latter results, in contrast to the data of Hall et al. [73], were obtained in non-specific patients from ICUs without selecting patients with severe AP.

Fluconazole at a dose of 400 mg per day is preferred to initiate antifungal prophylaxis in patients with a high risk of invasive candidiasis. Echinocandins are considered drugs of reserve in cases of fluconazole intolerance or high risk of fluconazole-resistant pathogens [71].

## Antibacterial and antifungal therapy in patients with infected necrosis

The advisability of using antibacterial drugs for infected necrosis is obvious, while timely diagnosis of infection presents some difficulties. In CT examination, the occurrence of gas is detected only in 40% of patients with infected necrosis [79]. Fine needle aspiration (FNA) can produce up to 12–25% false negative results and up to 14% false positive results [79, 80]. Frequent repeated tests during dynamic observation are associated with frequent radiation exposures; in addition, there is a risk of secondary infection via FNA. In current international practical guidelines, the routine use of FNA for the diagnosis of infected necrosis is not recommended [30, 32].

The serum level of procalcitonin ≥3.5 ng/ml for two consecutive days in patients with severe AP suspected for pancreatic infection has a sensitivity and specificity of 93 and 88%, respectively. However, this is true only for patients with multiple organ failure; in its absence, this indicator cannot serve as a marker of infection [81]. Despite the high sensitivity and specificity of the serum procalcitonin level, this test must be accompanied by a clinical examination because this parameter cannot be used by itself as the ultimate marker of infection [82]. Qu et al. [83], suggested using the serum procalcitonin level ≥0.5 ng/ml as an additional argument in favor of initiating antibiotic therapy in the presence of clinical signs of infection.

Currently, clinical signs in the form of worsening of the patient’s condition, intensification of pain, fever, and inflammation, in addition to laboratory confirmation of infection, are becoming increasingly important for choosing the treatment for suspected infected necrosis, including for starting antibiotic therapy [30, 32, 84]. With that approach, the preventive and therapeutic use of antibiotics cannot be clearly distinguished.

The choice of a specific antibiotic for suspected infected necrosis prior to microbiological verification of the pathogen and determination of its sensitivity is based on the known antimicrobial effect of this drug on the most frequent primary pathogens in pancreatic infection, which are gram-negative bacteria of the *Enterobacteriaceae* family and enterococci [50, 70, 72]. Carbapenems have the best (ultra-wide) antibacterial spectrum; moreover, ertapenem does not have clinically significant activity against *Pseudomonas aeruginosa* and thus does not contribute to the selection of multiresistant strains of this microorganism [85]. An important fact in favor of using carbapenems is the efficacy of this class of antibiotics as proven in two RCTs [20, 22].

The diagnosis of infected necrosis is an absolute indication for antibacterial drugs [86]. Despite the fact that the majority of patients with pancreatic infection require surgical intervention, the available clinical experience suggests that conservative treatment alone with carefully targeted antibacterial therapy will suffice in certain categories of patients [87–92]. The choice of antibiotic for infected necrosis is determined by the pathogenic bacterial agent and its sensitivity to antibiotics. In most cases, pancreatic infection is a mono-bacterial disease, with a transition from gram-negative to gram-positive microorganisms during the progression of the disease. In AP, extrapancreatic infections are commonly of polybacterial nature [49]. Infection with multidrug-resistant bacteria is an independent risk factor for death in patients with AP, which is a serious issue in practice of antimicrobial therapy [51, 70, 88, 93].

The use of continuous intravenous infusion of antibiotics is considered as an option to overcome the bacterial drug-resistance, however, no convincing evidence is presented regarding the beneficial outcome of this treatment in severe bacterial infections [94].

The administration of antibacterial drugs via regional intra-arterial infusion (RIAI) is aimed at improving their delivery to the pancreatic tissue; with intravenous administration the delivery is limited because of thrombosis and vasospasm that occur in necrotizing pancreatitis. To perform RIAI after CT with intravenous bolus contrast and angiography, the tip of the catheter is placed into the artery supplying the largest zone of hypoperfusion in the pancreas. In a number of studies, encouraging results of RIAI were observed: there was an increase in the survival rate with a significant decrease in the number of AP complications and the duration of hospitalization [95–97]. In contrast, Hamada et al. [98] found no reduction in mortality during RIAI; moreover, they had to treat purulent complications even more frequently. In a meta-analysis that covered 6 RCTs in 390 patients infused with antibiotics in combination with protease inhibitors, the efficacy of RIAI in patients with severe AP was substantiated [99]. Some of the international practical guidelines indicate that RIAI can be a therapeutic option in severe AP, but they give no direct recommendations for its use [30, 32]. In the Russian clinical guidelines, instructions for the intra-arterial administration of antibiotics are not mentioned [17].

The local use of antibacterial and antifungal drugs via a percutaneous catheter or endoscopic transmural drainage is indicated for increasing the local drug concentration in the focus of necrosis, where the diffusion of the antibiotic is the main factor determining its penetration into necrotic tissue. Using a mathematical model to calculate the diffusion coefficients, it was found that piperacillin, ceftriaxone, imipenem, gentamicin, ciprofloxacin, metronidazole, and vancomycin are able to easily diffuse into the pancreatic tissue. Imipenem shows the greatest empirical efficacy as it penetrates into deep tissue and preserves its activity for a long time; from this study, tigecycline and linezolid cannot be recommended for local treatment [100]. Several studies have demonstrated the efficacy of antibacterial and antifungal agents when applied topically, including the way of interstitial electrophoresis [3, 101, 102].

Another therapeutic option for topical application of antimicrobial agents is the use of an individually selected set of bacteriophages in patients infected with multiresistant strains; however, so far there are only a few reports on this technique [103].

## Conclusion

The present analysis of the recent literature indicates that the issue of antibacterial and antifungal therapy for acute pancreatitis remains unsolved. The results and conclusions of the reviewed studies are not consistent, which can be explained by the differences in study designs and the end points used (mortality, infection frequency, the need for surgical treatment and others). In addition, there are too few multicenter studies that would use identical criteria for patient characterization. Therefore, the decision to include antimicrobial agents in the therapeutic combination for acute pancreatitis cannot be generalized; it requires a balanced personalized and multidisciplinary approach.

The prophylactic use of antibiotics can be recommended primarily in patients with a high risk of infected necrosis, i.e., early organ failure, extended necrosis, intraperitoneal hypertension, dynamic intestinal obstruction, or severe immunosuppression in the early phase of the disease (assessed by low lymphocyte counts). Independent high-risk factors also include the biliary etiology of acute pancreatitis and portosplenomesenteric venous thrombosis detected by CT, as well as abnormally high values of the following laboratory parameters obtained during the first 48 h: hematocrit ≥50%, urea ≥8.42 mmol/L, C-reactive protein ≥257 mg/L, and procalcitonin level ≥1.39 ng/ml.

Another aspect of antibacterial prophylaxis is its use in patients with clinical signs of infection (fever, persisting pain, a shift in the leukocyte formula). According to the current practical recommendations, in suspected infected necrosis, fine-needle aspiration or repeated CT are no more mandatory; the medical team is advised to focus on patient’s clinical status, instead [30, 32]. For the preventive use, it should be borne in mind that only carbapenems have been conformed for their capability of reducing the incidence of pancreatic necrosis. Selective intestinal decontamination can be effective for the prevention of necrosis infection in patients with severe acute pancreatitis, but there is no sufficient evidence to recommend this treatment.

The role of antifungal prophylaxis remains undetermined. None of the current practical guidelines provide guidance on its application to patients with acute pancreatitis. It seems appropriate to prescribe antifungal drugs to patients with a high risk of developing invasive candidiasis, which can be evaluated using the Candida Score, Modified Invasive Candidiasis Score, or Candida Colonization Index Score. It is advisable to specifically characterize patients with a high risk of candidiasis by using non-cultural serological diagnostic tests for antibodies to the growth tubes of *Candida albicans*, beta-1,3-D-glucan, mannan antigen, and antibodies to mannan [77, 104]. Fluconazole is preferred for antifungal prophylaxis in the absence of known resistance to this drug in a particular ICU.

Antimicrobial therapy of infected necrosis should be accompanied by repeated microbiological testing of the material from the necrosis zones and from the oropharynx, bronchial tree (sputum, discharges from the endotracheal tube or tracheostomy cannula), urinary tract, and blood to monitor possible pancreatic and extrapancreatic infections, and to detect early signs of multi-resistance. To overcome the bacteria resistance to antibiotics, alternative routes of administration of antibacterial and antifungal agents can be used: i.e., prolonged intravenous infusion, intra-arterial infusion, or local administration via an inserted catheter.

The above issues concerning antibacterial and antifungal therapy in patients with AP emphasize the problems that need to be solved in further studies.
